# Conscious monitoring and control (reinvestment) in surgical performance under pressure

**DOI:** 10.1007/s00464-012-2193-8

**Published:** 2012-02-21

**Authors:** Neha Malhotra, Jamie M. Poolton, Mark R. Wilson, Karen Ngo, Rich S. W. Masters

**Affiliations:** 1 Institute of Human Performance, University of Hong Kong, Hong Kong, Hong Kong; 2 Department of Surgery, University of Hong Kong, Hong Kong, Hong Kong; 3 School of Sport and Health Sciences, College of Life and Environmental Sciences, University of Exeter, Exeter, EX1 2LU UK; 4 Institute of Performance, The Hong Kong Jockey Club Building for Interdisciplinary Research, 3/F, 5 Sassoon Road, Pokfulam, Hong Kong, Hong Kong

**Keywords:** Reinvestment, Laparoscopic training, Motor skills, Time pressure, Surgical stressors, Motor learning and control

## Abstract

**Background:**

Research on intraoperative stressors has focused on external factors without considering individual differences in the ability to cope with stress. One individual difference that is implicated in adverse effects of stress on performance is “reinvestment,” the propensity for conscious monitoring and control of movements. The aim of this study was to examine the impact of reinvestment on laparoscopic performance under time pressure.

**Methods:**

Thirty-one medical students (surgery rotation) were divided into high- and low-reinvestment groups. Participants were first trained to proficiency on a peg transfer task and then tested on the same task in a control and time pressure condition. Outcome measures included generic performance and process measures. Stress levels were assessed using heart rate and the State Trait Anxiety Inventory (STAI).

**Results:**

High and low reinvestors demonstrated increased anxiety levels from control to time pressure conditions as indicated by their STAI scores, although no differences in heart rate were found. Low reinvestors performed significantly faster when under time pressure, whereas high reinvestors showed no change in performance times. Low reinvestors tended to display greater performance efficiency (shorter path lengths, fewer hand movements) than high reinvestors.

**Conclusion:**

Trained medical students with a high individual propensity to consciously monitor and control their movements (high reinvestors) displayed less capability (than low reinvestors) to meet the demands imposed by time pressure during a laparoscopic task. The finding implies that the propensity for reinvestment may have a moderating effect on laparoscopic performance under time pressure.

Surgeons are required to execute highly specialized skills in safety critical environments in the presence of a variety of intraoperative stressors [1, 2]. Although validated curricula have been developed to train technical surgical skills, the potentially negative impact that acute stress has on surgical performance has been relatively ignored [3, 4]. In other safety critical domains, such as aviation and anesthesiology, this has not been the case [3, 5].

Potential stressors that can disrupt the technical and decision-making components of surgical performance in simulated [2, 6] and operating room (OR) environments [2] include lack of experience [7–9], procedural complexity [7], time pressure [10–12], and distractions [10] (see [4] for review). In the domain of surgery, few studies have investigated the cognitive mechanisms that underlie the disruptive effects of these stressors; however, an extensive body of work in the domain of motor learning has discussed the underlying cause of disruptions to specialized motor skills [13–15]. Theoretical principles established in the motor-learning domain should, therefore, serve as a useful resource to inform surgical education.

Two cognitive processes, distraction and self-focus, are considered to be the primary contributors to motor skill disruption under stress. Stress can distract the attention of a performer away from relevant aspects of a task [16–18], or, alternatively, stress can cause attention to be directed toward movement, a process described by the theory of reinvestment (see [19] for a review). The theory argues that contingencies such as psychological stress can cause performers to make conscious efforts to ensure the quality of performance by monitoring (movement self-consciousness) and controlling (conscious motor processing) their movements. As a result, components of the skill that ordinarily are executed automatically are disrupted, the fluidity of the movement is lost, and performance breaks down [20]. In other words, conscious efforts may ironically lead to suboptimal performance. Any intraoperative stressor that is sufficiently acute to cause surgeons to reinvest may potentially disrupt performance of technical skills and lead to error.

The likelihood that reinvestment will occur in response to stressors has been shown to be dependent not only on the severity of the stressor but also on individual personality differences [21–23]. An individual’s predisposition toward reinvestment, and, therefore, the susceptibility to skill breakdown, can reliably be quantified by completion of a Movement-Specific Reinvestment Scale [23]. Reinvestment scores have been shown to correlate with negative performance change due to the introduction of psychological stressors [13, 21–24].

The possibility that reinvestment plays a role in surgical performance under stress has previously been mentioned in the surgical literature [25], but this study is the first to investigate whether an individual’s propensity for reinvestment moderates the impact of a common intraoperative stressor (time pressure [10–12]) on performance of a laparoscopic task.

## Method

Thirty-seven undergraduate medical students (years 4–5) from the University of Hong Kong volunteered to take part in the study. To ensure that prior laparoscopic training did not confound the findings, medical students with no prior laparoscopic experience were recruited. Six of the participants eventually withdrew from the study due to scheduling constraints. Ethical approval for the study was obtained from the Institutional Review Board. All participants provided written informed consent and completed the Movement-Specific Reinvestment Scale (MSRS) prior to participation [23]. The MSRS comprises ten items that relate to concerns about the style of movement (e.g., “I am self conscious about the way I look when I am moving”), and conscious attention to the process of movement (e.g., “I am aware of the way my body works when I am carrying out a movement”). Participants rated each item on a 6-point Likert scale from “strongly disagree” to “strongly agree.” Thus, cumulative scores ranged from 10 to 60 points, with high scores indicating individuals with a high propensity for reinvestment. The MSRS has high test–retest and internal reliability [19] and has informed research on clinical and nonclinical populations [26–28].

The experimental procedure comprised a training session and a test session. Participants attended sessions individually. They trained on the laparoscopic peg transfer task, a manual skills component of the Fundamentals of Laparoscopic Surgery (FLS) training module, developed by the Society of American Gastrointestinal and Endoscopic Surgeons (SAGES) and endorsed by the American College of Surgeons [29]. The task was completed on an FLS laparoscopic trainer box. Participants were required to use grasper forceps to transfer and position six triangular plastic objects (one at a time) from one side of a pegboard to the other and back again. For the first half of the trial the pegs were transferred from the nondominant hand to the dominant hand and for the second half of the trial the process was reversed. The task was timed and trials in which pegs were dropped out of reach or out of the field of view of the camera were discounted.

An instructional video was shown to participants before training commenced. Training ended when participants achieved a predetermined proficiency level, defined by the developers of the FLS training module [29] as task completion within 54 s on two consecutive trials followed by an additional ten trials at criterion level. Participants were aware of the criterion level and were provided feedback upon request. A rest was allowed after every ten trials or more frequently if required. Fifteen of the participants reached criterion proficiency levels within one 90-min session. Sixteen participants returned for an additional training session within 5 days, dependent on the time constraints of the participants and the laboratory.

No more than 48 h after reaching proficiency, participants returned for the test session. First, participants familiarized themselves with the task until they completed consecutive-criterion level trials. They then performed two trials in a control condition and two trials in a time pressure condition. The two conditions were counterbalanced to avoid order effects. In the control condition, participants were simply asked to do their best, as they had in training. In the time pressure condition, participants were informed that operating surgeons sometimes are required to perform under time constraints (e.g., trauma) and on the upcoming trials they should try to complete the task faster than their best time in training (of which they were informed). Following the test session, participants were fully debriefed.

To assess the impact of the time pressure manipulation on the stress levels of participants, two of the three measures of the Imperial Stress Assessment Tool (ISAT) [30] were employed. Heart rate was recorded using a Polar S810 (Polar Electro Oy, Kempele, Finland) monitor from the start of each trial until the last object was placed. Average heart rate in each condition was used as the dependent variable [31, 32]. The State Trait Anxiety Inventory (STAI, short version [33]) was completed after each condition. The STAI consists of six statements (I feel calm; I feel tense; I feel upset; I am relaxed; I am content; I am worried), which required a Likert scale response (1 = not at all to 4 = very much so) with regard to the last two trials that the participants had completed.

Performance outcome was assessed by completion time and number of object drops in each trial [34]. As a process tracing measure of how time pressure might influence performance, hand movements were recorded for each trial using the Imperial College Surgical Assessment Device (ICSAD). The dorsum of each hand was fitted with a motion-tracking sensor (Isotrak II, Polhemus, VT) and position data were processed through a Gaussian filter and converted into path length and number of movements using proprietary software [35, 36]. Path length (the combined total path travelled by each hand in *x*, *y*, and *z* coordinates) and number of movements were used as dependent measures [31, 32].The motion-tracking sensors and the heart rate monitor were worn throughout training and during the test session.

### Statistical analysis

Pearson product moment correlations were first computed to examine the general association between MSRS scores and changes in completion time, number of object drops, path length, and number of movements because of time pressure (Δ = time pressure − control). Further analysis was conducted by separating participants into groups of high and low reinvestors using a median split. The median split of the 31 participants resulted in 12 high reinvestors and 15 low reinvestors (4 participants had the median score of 41; range = 24–59).^1^ An independent-samples *t* test confirmed that the mean score for the low reinvestors (34.2 ± 1.15) and the high reinvestors (47.25 ± 1.42) differed significantly (*p* < 0.001). On the basis of the median split, mixed-design Group (low reinvestors, high reinvestors) × Condition (control, time pressure) analyses of variance (ANOVA) were computed. Follow-up *t* tests were used to explain the interaction effects where appropriate.

## Results

### Training

Participants took on average 58.04 ± 4.03 trials to reach proficiency in the training phase of the study. Low reinvestors and high reinvestors did not significantly differ in the number of trials required to reach proficiency (61.27 ± 5.68 vs. 54.00 ± 5.70, respectively; *p* = 0.381). Furthermore, the best training times of low reinvestors and high reinvestors did not differ significantly (42.40 ± 0.65 s vs. 41.00 ± 0.75 s, respectively; *p* = 0.170).

### Testing

#### Stress measures

**Fig. 1:**
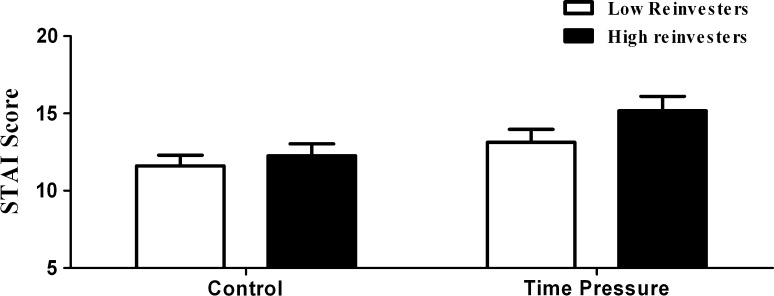
Subjective measure of anxiety (STAI score) of high and low reinvestment groups across control and time pressure conditions

The analysis of variance for STAI scores revealed a significant effect of Condition (*p* < 0.001) but no significant effect of Group (*p* = 0.208) and no significant interaction (*p* = 0.184). As shown in Fig. 1, STAI scores were significantly higher in the time pressure condition than in the control condition (14.04 ± 0.64 vs. 11.89 ± 0.51, respectively). The analysis of variance for the heart rate data revealed no significant effects of Condition (*p* = 0.248) or Group (*p* = 0.444) with no significant interaction (*p* = 0.639). The participants’ heart rate data revealed no significant differences between the control condition (87.18 ± 2.57) and the time pressure condition (88.30 ± 2.80).

### Performance outcome

#### Completion time and number of drops

**Fig. 2:**
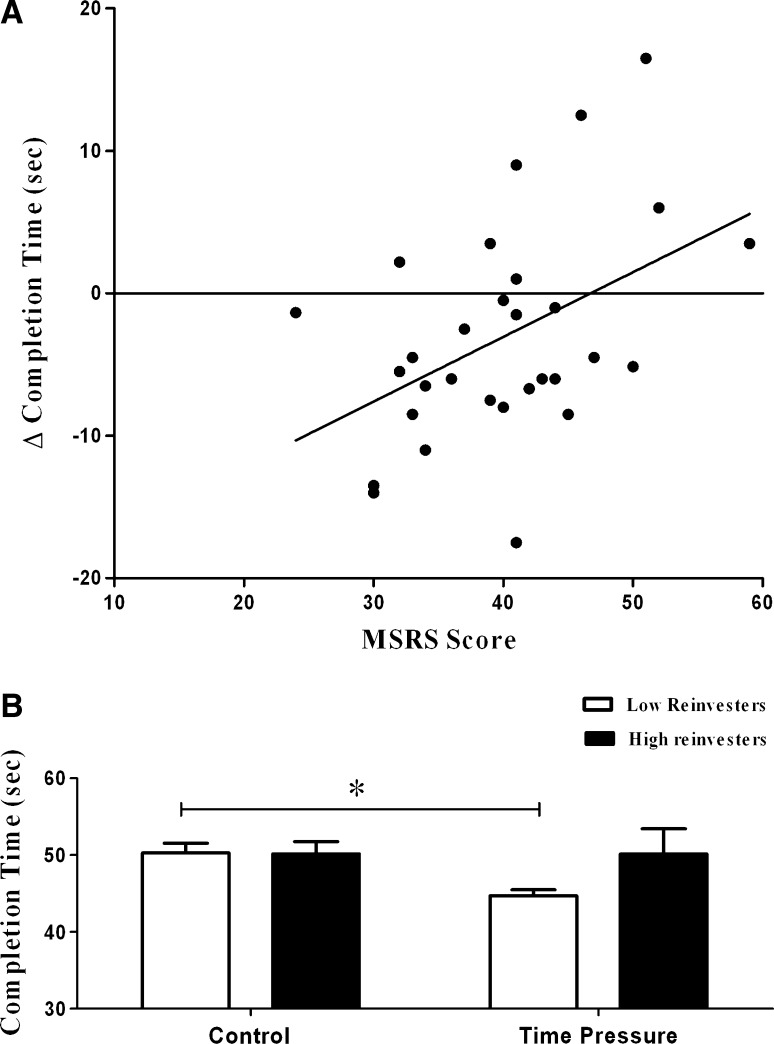
**A** Correlation between performance data (Δ completion time = time pressure − control) and MSRS. **B** Performance measured as completion time(s) of high and low reinvestment group across control and time pressure conditions

Correlational analysis revealed that MSRS scores were positively correlated with change in completion time from the control to the time pressure condition (*r* = 0.46, *p* = 0.010), explaining 20.7% of the variance (see Fig. 2A).

Analysis of the median split data for completion time revealed no significant effect of Group (*p* = 0.257), but a significant effect of Condition (*p* = 0.038) was present with a significant interaction (*p* = 0.040). As shown in Fig. 2B, low reinvestors displayed significantly reduced completion times from the control condition to the time pressure condition (*p* = 0.001), while high reinvestors displayed no significant change (*p* = 0.990).

Due to the minimal number of drops in the retention (0.33 ± 0.08) and the time pressure (0.26 ± 0.10) conditions, statistical analysis was not conducted on this performance outcome measure.

### Process tracing measures

#### Path length and number of movements

Correlational analysis revealed that MSRS scores were not significantly correlated with change in path length from the control to the time pressure condition (*r* = 0.345, *p* = 0.057), although the effect approached significance, explaining 11.9% of the variance.

**Fig. 3:**
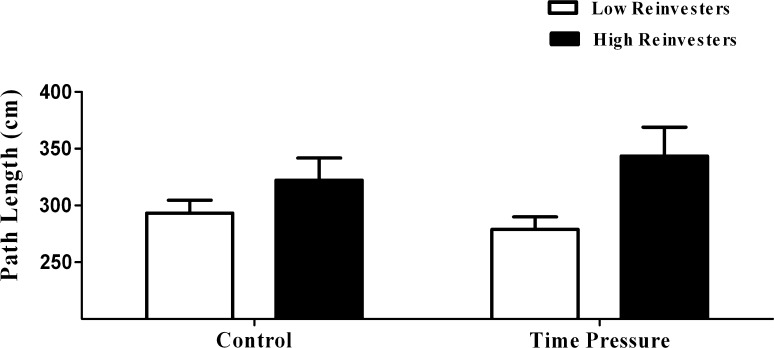
Performance measured as path length (cm) of high and low reinvestment group across control and time pressure conditions

Analysis of the median split data for path length revealed no significant effect of Condition (*p* = 0.654), but a significant effect of Group was present (*p* = 0.048) along with a significant interaction (*p* = 0.028). Figure 3 indicates that low reinvestors (286.02 ± 15.00) tended to have shorter path lengths compared to high reinvestors (332.91 ± 16.77) and they tended to display reduced path lengths from the control to the time pressure condition, whereas high reinvestors tended to display increased path lengths from the control to the time pressure condition, although these effects were not significant (*p* = 0.100 and *p* = 0.149, respectively).

Correlational analysis revealed that MSRS scores were not significantly correlated with change in number of movements from the control to the time pressure condition (*r* = 0.293, *p* = 0.110).

Analysis of the median split data for number of movements revealed neither a significant effect of Condition (*p* = 0.234) nor an interaction effect between Group and Condition (*p* = 0.389), with only the effect of Group approaching significance (*p* = 0.060). Low reinvestors (41.42 ± 1.28) tended to make fewer movements than high reinvestors (45.19 ± 1.43).

## Discussion

Previous studies have identified potential stressors that can be detrimental to laparoscopic performance [7–12]. Most have focused on external factors that affect performance rather than internal mechanisms that underpin poor performance under stress [4]. This study set out to investigate whether an individual’s propensity to consciously monitor and control movement (or reinvest) moderated performance under a common intraoperative stressor: time pressure.

The time pressure manipulation heightened the importance of completing an operation quickly and increased trainees’ perceived anxiety. Under these conditions, trainees categorized as low reinvestors were better able to meet the task demands by quickening their completion time than those categorized as high reinvestors. Overall, the findings are consistent with previous research outside the surgical domain that has shown a relationship between reinvestment and performance under pressure [13, 21–24].

According to the theory of reinvestment, higher reinvestment scores reflect an increased tendency for an individual to focus attention inward in an attempt to consciously monitor and control movements, especially in anxiety-provoking conditions [19]. Consequently, we expected that time pressure would disrupt path lengths and the number of hand movements of high reinvestors because they would be more likely to deploy conscious monitoring and control during performance of the FLS task [13, 28, 37]. However, our data show that high reinvestors had longer path lengths and more hand movements than low reinvestors, regardless of whether they were under time pressure. Thus, although both high and low reinvestors reached the standardized proficiency level (trial completion within 54 s) within a similar number of training trials, the low reinvestors were more efficient.

The presence of a group (high reinvestors, low reinvestors) × condition (control, time pressure) interaction for path length suggests that there was a trend toward more efficient performance under time pressure (shorter path lengths) by low reinvestors and less efficient performance under time pressure (longer path lengths) by high reinvestors, but neither group showed a significant change in performance efficiency from control to time pressure conditions. Why then did high reinvestors not demonstrate quicker performance times under time pressure? High scores on the Movement-Specific Reinvestment Scale reflect not only a greater propensity to consciously control movements, but also greater propensity to monitor the style or form of the movements that have been learned [23]. Even under conditions that explicitly demanded a quickening of movement, high reinvestors may have prioritized the style or form of their laparoscopic movements over speed.

Psychological stress is the most obvious contingency that induces reinvestment [19]. Moderate levels of psychological stress may lead to enhanced performance, but when the demands of a task outweigh available coping resources, an individual may feel the need to control the situation by consciously monitoring and controlling performance [19, 38]. Our findings imply that reinvestment may have a moderating effect on performance under one of the many psychological intraoperative stressors, time pressure, but it is possible that a predisposition to reinvest may have a moderating impact on the effects of other disruptive contingencies present in the surgical environment as well (e.g., sleep deprivation and physiological fatigue).

Although the study suggests a moderating role of reinvestment on performance of technical skills under stress, it is yet to be seen whether this effect extends to nontechnical facets of laparoscopic performance, such as decision-making. Operative surgery requires the surgical trainee to exhibit not only sound technical skill but also timely decision-making [39]: the surgeon’s scalpel is said to be “the tip of an ever changing and evolving decision making process” [40, p. 98]. Recent studies have extended the association between reinvestment and skilled performance from the motor skill domain to cognitive tasks involving decision-making components [14]. Subsequently, a decision-specific version of the Reinvestment Scale [41] has been developed based on the Masters and colleagues version [23]. The decision-specific Reinvestment Scale will enable more precise investigation of the association between reinvestment and decision-making in surgical tasks.

One way to combat reinvestment is to train skills using implicit motor-learning techniques [20, 37, 42], which reduce the opportunity to gain movement-specific verbal knowledge yet allow acquisition of the technical competence required for skill execution. By reducing the likelihood of reinvestment, implicit motor learning has been shown to result in performance that is robust under psychological pressure [20, 21], physiological fatigue [43, 44], multitasking [45, 46], and time pressure [47]. Implicit motor learning has been suggested as an alternative theoretical framework [48] for training surgical skills. Preliminary work that has pioneered implicit motor learning in surgery has shown some promise in this avenue [49, 50]. For example, Zhu and colleagues [50] claimed that implicit motor learning promotes greater neural efficiency in a laparoscopic task, which may allow surgeons to better cope with challenges such as stress, fatigue, and complex decision-making.

This study was an initial attempt to investigate the underlying mechanisms that contribute to performance in the presence of intraoperative stressors. Psychological stress is a constant factor in the OR but potential to cope with psychological stress is adjusted by individual differences, as illustrated by our results and those from the motor-learning domain [21–23]. The study serves as a departure point for further investigation of the potential moderating impact of reinvestment on surgical performance in real world settings (OR), in more complex surgical tasks, and across levels of expertise. However, it is probably too early to claim that the capacity to assess an individual’s propensity for movement-specific reinvestment is useful for screening surgical aptitude. Rather than screening, it may be preferable to modify reinvestment as a trait. The propensity to reinvest has been shown to increase as a consequence of duration of Parkinson’s disease [26], so it may well be possible to develop surgical training interventions designed to reduce the propensity for reinvestment. For example, implicit motor learning, as discussed above, may serve this purpose.

Future studies should also explore the association between this individual predisposition and other psychological stressors associated with technical skill error, as well as cognitive aspects involved in achieving operative excellence, such as decision-making. The findings from this study and prospective studies can inform the development of curricula that can be tailored to the needs of the individual.

## Footnotes

1 Based on previous studies in the motor skill learning domain [13, 28] a post-hoc median split on 31 participants (leading to 12 high reinvestors and 15 low reinvestors) provided sufficient power for the study.
